# Editorial: Renal injury and the brain

**DOI:** 10.3389/fmed.2022.1100487

**Published:** 2023-01-04

**Authors:** Caroline G. Shimoura, Kedra Wallace, Keisa W. Mathis

**Affiliations:** ^1^Department of Physiology and Anatomy, University of North Texas Health Science Center, Fort Worth, TX, United States; ^2^Department of Pharmacology, University of Mississippi Medical Center, Jackson, MS, United States

**Keywords:** acute kidney injury, chronic kidney disease, renal injury, kidneys, brain

Acute kidney injury (AKI) is a rapid and reversible decline in renal function accompanied by increased urinary albumin. The list of causative factors for AKI is extensive, but includes decreased perfusion to the organ, parenchymal disease that can affect both structure and function, as well as obstructive causes. Chronic kidney disease (CKD) is the persistent and progressive impairment in kidney function and/or kidney injury for at least 3 months (1). While CKD can develop through similar causes as AKI, it is most commonly due to hypertension and diabetes. According to the Center for Disease Control and Prevention, CKD affects over 37 million people in the United States and is associated with high morbidity and mortality; yet, nine out of 10 of these people are unaware they have the disease (2). So, determination of new approaches to early diagnostics in addition to improved awareness of AKI and CKD are critical to halt disease development and avoid the need for extreme interventions such as dialysis and renal transplant.

Declining renal function is associated with neurological disorders (3–5). In fact, it has been reported that AKI patients have altered cerebral blood flow due to neurovascular dysfunction and other neurological abnormalities (6) and 60% of CKD patients have impaired cognitive function (7). Both neurological and renal diseases are worldwide public health problems that often have complex medical issues and might lead to detrimental consequences; therefore, studies to confirm mechanistic links are warranted. Many question whether neural dysfunction promotes renal inflammation, structural abnormalities, hypertension, leading to AKI and CKD, or vice versa? The goal of this Research Topic is to elucidate potential mechanisms involved.

Many efforts have been made to predict kidney disease. For example, Chen et al. used available datasets from the Medical Information Mart for Intensive Care (MIMIC) study to test a new attention-based temporal neural network approach to predict AKI cases, stage of disease, and onset time. With this approach, it will be possible to detect AKI in early stages, before the onset of the kidney injury. They verified that factors as such lactate, glucose, creatinine, blood urea nitrogen, prothrombin time, and partial thromboplastin time are positively correlated with AKI, while platelet count, hemoglobin levels, and hematocrit are negatively correlated. Interestingly, Wu et al. showed that other conditions such as exertional heatstroke caused by an increase in core body temperature by intense physical activity, can lead to AKI-associated disability and death. This study used a compilation of clinical risk factors including reduced blood volume, microthrombi in renal afferent and efferent arteries, blockage of the renal tubules, nephrotoxicity, and renal interstitial cell inflammation to characterize renal disease with neurological dysfunction for better diagnosis. Future studies must continue to establish associative symptoms and parameters to allow early diagnosis, which may help improve treatment and avoid disease severity progression.

Patients with CKD are at a higher risk for developing cognitive impairment, so late diagnosis can decrease a patient's quality of life and affect memory, attention, and executive function (cognitive control and behavior). Adherence to treatment may be beneficial (8); in fact, studies show that patients that underwent kidney transplantation showed improvement in cognitive function, specifically in psychomotor speed, attention, visual planning, learning, and memory (9). The rationale is that CKD patients have an impairment in cerebral blood blow and vascular damage as well as accumulation of uremic toxins, oxidative stress and inflammation, and this can all promote neurological disorders (6, 10). This phenomenon may be extended to explain the link between post-partum AKI and cognitive dysfunction. Szczepanski et al. hypothesized that the increase in oxidative stress and inflammatory factors leads to the impairment in blood brain barrier and consequently, neuroinflammation and cognitive disorder in such patients. AKI in post-partum can have long term consequences in renal, cardiovascular and central nervous systems (Gupta et al.). As patients with hypertension and thrombotic microangiopathic disorders during pregnancy are predispose to AKI post-partum, we can expand Szczepanski et al. hypothesis in the context of other pathologies.

Using an autoimmune disease model of systemic lupus erythematosus (SLE), Morales et al. studied the link between hypertension, inflammation and cognitive impairment. Although their hypothesis that the activation of the cholinergic anti-inflammatory pathway at the level of nicotinic receptors using systemic administration of positive allosteric modulators, also called PAMs, would improve renal disease and cognitive disorder in SLE mice with advance disease was not confirmed, the rationale that increased blood pressure and inflammation leads to cognitive impairment corroborates with Szczepanski et al. and others. Also, Renczes et al. could not link kidney injury with cognitive impairment and locomotor activity deficit in rats. The aim of their study was to better understand the development and maintenance of cognitive impairment in CKD over the course of CKD using the 5/6 nephrectomy model. They analyzed locomotor activity and cognitive behavior in rats in two different time points of the disease. Also, markers of kidney function and injury were analyzed to track the progression of CKD. Although, CKD rats presented impaired kidney function as early as 2 months there was no significant changes in behavior and locomotion activity during the study, contradicting other studies (11–13). Reasonably, the author points out that the data presented show high variability and fluctuation over time. Unfortunately, no correlation analysis was done in order to identify association between the level of kidney injury and function with behavior and locomotor function. Furthermore, many studies hypothesize that the cause for cognitive dysfunction in CKD is due to an impairment in cerebral blood blow and vascular damage (10), but neither this nor blood pressure were measured in this study. Together, these two studies suggest that the link between kidney disease, inflammation and cognitive impairment are complex and future studies using different models, different stages of the disease and other targets will be able to untangle this hypothesis.

Besides disorders related to the cardiovascular system, conditions as Alzheimer's disease have also been associated with CKD. Cystatin C is a cysteine protease inhibitor that is freely filtered by the glomerulus and degraded in the proximal tubule. It is used as a biomarker of kidney function since the accumulation is an estimative of glomerular filtration rate. Studies show that amyloid-b, a component of amyloid plates responsible for Alzheimer's disease, co-localize with cystatin C within the brain. Lau et al. and Lau et al. (14) not find association between brain amyloid-b and cystatin C-estimated glomerular filtration rate (eGFR) but in a previous study they did find that an association between cognition and central nervous system microvasculature in the same cohort of patients. Also, Zemp et al. observed that physical impairment in gait quality caused by damage to the central nervous system precedes cognitive dysfunction in older patients with CKD. The authors verified that pre-dialysis and dialysis patients have gait movement disorder and the early diagnosis with simple and non-invasive techniques can allow preventive treatment/early interventions despite further central nervous system complication caused by CKD.

The strong relationship between neurological disorders and renal injury can be explained by the fact that both the brain and the kidneys share similar hemodynamic characteristics and are highly susceptible to vascular damage (4). Understanding the pathophysiology of the risk factors of kidney injury including inflammation, hypertension and other cardiovascular diseases are extremely important to better treat kidney disease and prevent further complications with central nervous system processes.

## Author contributions

CS, KW, and KM drafted, edited and revised the manuscript, and agreed with the final version.
